# Reducing Ex-offender Health Disparities through the Affordable Care Act: Fostering Improved Health Care Access and Linkages to Integrated Care

**DOI:** 10.3934/publichealth.2014.2.76

**Published:** 2014-04-18

**Authors:** Lacreisha Ejike-King, Rashida Dorsey

**Affiliations:** U.S. Department of Health and Human Services, Office of Minority Health, 1101 Wootton Parkway Suite 600, Rockville, MD 20852, USA

**Keywords:** Incarceration, reentry population, health disparities, Affordable Care Act, racial and ethnic minorities

## Abstract

Despite steadily declining incarceration rates overall, racial and ethnic minorities, namely African Americans, Latinos, and American Indians and Alaska Natives, continue to be disproportionately represented in the justice system. Ex-offenders commonly reenter communities with pressing health conditions but encounter obstacles to accessing care and remaining in care. The lack of health insurance coverage and medical treatment emerge as the some of the most reported reentry health needs and may contribute to observed health disparities. Linking ex-offenders to care and services upon release increases the likelihood that they will remain in care and practice successful disease management. The Affordable Care Act (ACA) offers opportunities to address health disparities experienced by the reentry population that places them at risk for negative health outcomes and recidivism. Coordinated efforts to link ex-offenders with these newly available opportunities may result in a trajectory for positive health and overall well-being as they reintegrate into society.

## 1. Introduction

Nearly seven million individuals residing in the United States were in jail or prison or on probation or parole at the end of 2012 [1]. U.S. prisons discharge approximately 700,000 inmates annually [2]. Incarceration and release statistics indicate overrepresentation of racial and ethnic minorities. Post-release, ex-offenders face a host of reintegration barriers including decreased civic participation, economic instability, housing unavailability, under-education and unemployment [3]. Ex-offenders commonly reenter communities with pressing health conditions but encounter obstacles to accessing care and remaining in care. The purpose of this report is to discuss how new health care coverage opportunities made available by the Patient Protection and Affordable Care Act (ACA) and utilization of integrated health care models may reduce health disparities experienced by the reentry population. Strategies for linking reentrants to such newly available health care coverage opportunities and to integrated care services will also be discussed.

## 2. Minorities and the justice system

Despite steadily declining incarceration rates overall, racial and ethnic minorities, namely African Americans, Latinos, and American Indians and Alaska Natives (AI/AN), continue to be disproportionately represented in the justice system. Together, African Americans and Latinos comprise 60% of the prison population [4] despite representing 30% of the U.S. population [5]. Both groups experience incarceration rates higher than Whites [6],[7], a disparity most pronounced among males. Approximately 1 in 3 African American males and 1 in 6 Latino males will be imprisoned during their lifetime, as compared to 1 in 17 whites [7]. Though faring better than males, African American females and Latinas also experience incarceration at rates higher than their White counterparts [6]. Additionally, African Americans and Latinos combined were nearly 60% of the paroles in 2011 [8].

Approximately 79,000 AI/ANs were incarcerated in 2010 [9]. Though AI/ANs represented only 1.4% of the U.S. inmate population in 2010, state-specific data identifies great disparities in the state incarceration rates of AI/AN populations. For example, in South Dakota, AI/ANs represent about nine percent of the state's population [5], but comprise over 27% of the adult incarcerated population [10]. Overall, the AI/AN incarceration rate in the U.S. is higher than that reported for Whites [6].

## 3. Health disparities among justice-involved persons

Medical care is available to individuals while incarcerated; however, physical and behavioral health conditions remain prevalent among justice involved populations. Many inmates with communicable diseases such as tuberculosis, Hepatitis B, Hepatitis C and HIV/AIDS pass through the correctional system annually [11],[12]. The rates of these conditions greatly surpass those of the total U.S. population, and ex-offenders with these conditions comprise a sizeable portion of those diagnosed in the U.S. [12]. Justice-involved individuals also experience chronic conditions that mirror growing health problems witnessed throughout the U.S., such as hypertension, asthma and diabetes [12]. Estimates indicate that 800,000 U.S. prisoners experience at least one chronic condition [13]. Disparities exist in the rates of these chronic diseases in the U.S. prison population when compared to the general U.S. population [13].

Mental illness and substance abuse also pose serious health risks among the justice-involved population. Large proportions of incarcerated individuals have mental health problems [14]. The rate of mental illness among the justice-involved population is estimated to be higher than that of the general U.S. population [12],[13]. For example, the reported prevalence of schizophrenia/ psychosis disorder among state inmates was triple the lifetime prevalence reported for the U.S. population [12]. Mental illness and substance use disorder issues often exist as comorbidities. Among justice-involved individuals, half of all inmates have abused alcohol or drugs. These rates are elevated among those with mental illness [14]. Unfortunately, many reentrants engage in pre-incarceration behavior patterns leading to resumed substance use shortly after release [15].

National health disparities data based on race or ethnicity within the justice-involved community is limited and warrants attention [16],[17]. The existent sparse disaggregated data indicates substantial substance use disorder and mental illness occurrence [14], higher AIDS death rates [18], and higher prevalence of sexually transmitted diseases, tuberculosis and HIV [19] among African American and Latino inmates. The AI/AN population possesses high rates of substance-related offenses [20],[21] that may indicate disparate rates of AI/AN substance abuse disorders among inmates. Though there is an incomplete understanding of racial and ethnic health disparities experienced within criminal justice populations, the assumption of health inequities is strengthened by general population disparities data. Minorities in the general U.S. population experience disproportionate rates of morbidity and mortality from many of the same infectious and chronic conditions experienced by the justice-involved population.

## 4. Health care access and maintenance issues among the reentry population

Upon release, ex-offenders contend with many competing priorities to reestablish their lives, oftentimes with health care taking a lower priority to more pressing survival needs such as food and housing. However, the lack of health insurance coverage and medical treatment emerge as the most reported reentry health needs and may contribute to observed health disparities [22]. The health and behavioral health conditions mentioned in the previous section are often exacerbated by the lack of health care accessibility post-release. A substantial proportion of individuals involved with the criminal justice system do not have health insurance coverage [23]. In most states, those receiving Medicaid benefits prior to incarceration face termination of their eligibility as a result of their imprisonment [24]. The post-incarceration Medicaid reapplication process disrupts continuity of care for ex-offenders through restricting access to necessary health care services.

Many ex-offenders are released to a health care system that has not been trained to be responsive to populations with co-occurring medical, psychological and substance dependency issues. Unable to find appropriate care, offenders with chronic conditions may relapse or worsen. For those with mental and substance use disorders, disruption in treatment may lead to reoffending.

## 5. The Affordable Care Act and opportunities to improve reentrant health

The Affordable Care Act (ACA), signed into law in 2010, promotes accessible, quality and affordable health care for Americans and is expected to contribute to the reduction of health disparities experienced by vulnerable populations [25]. ACA provisions establish avenues to increase health care accessibility through health insurance coverage availability, specifically the expansion of Medicaid eligibility and the creation of the Health Insurance Marketplace (Marketplace). Medicaid eligibility will extend to low-income and nonelderly individuals without children below 133% of the federal poverty level in states that choose to expand their Medicaid program. Individuals not eligible for Medicaid may qualify for subsidies to help pay insurance premiums for private health plans selected in the Marketplace. One national simulation estimated that 34% of ex-offenders will be eligible for Medicaid and an additional 24% of reentrants will be eligible for subsidies within the Marketplace [26]. However, smaller scale studies in state and local jurisdictions have put the rates much higher. For example, New York City estimates that 89% of their correctional population will be eligible for Medicaid [26] and the State of Michigan estimates that 80% of their prison inmates will be eligible [27]. In addition to financial assistance, all private health insurance plans offered in the Marketplace will, at a minimum, cover 10 essential health benefits (Box 1). Enrolling in public or private health insurance coverage increases the likelihood that reentrants will receive care for chronic medical, mental and behavioral issues. This has the potential for reducing disparities in health care access and health outcomes between the criminal justice and general population.

**Essential Health Benefits**

1. Ambulatory patient services

2. Emergency services

3. Hospitalization

4. Maternity and newborn care

5. Prescription drugs

6. Rehabilitation and habilitative services and devices

7. Laboratory services

8. Preventive and wellness services and chronic disease management

9. Mental health and substance use disorder services, including behavioral health treatment

10. Pediatric services, including oral and vision care

ACA provisions support integrated health care approaches for persons with chronic comorbidities [25]. The Affordable Care Act introduces “health homes” as viable options for addressing and managing the complex health needs of those individuals. Health homes promote a holistic approach to health care by providing medical and behavioral care coordination and management in addition to providing support for patients and their families, and linkages to community services for individuals with multiple chronic conditions or risk of developing additional chronic conditions [28]. Community health centers (CHCs), long lauded for the promotion and provision of coordinated, primary care for indigent and medically underserved communities, have received additional funds under the ACA for operation, expansion and construction [29] and will likely have a continual influence on the care for low-income individuals ineligible for Medicaid [30]. The Innovation Center at the Centers for Medicare & Medicaid Services (CMS Innovation Center) was established by the ACA for the purpose of testing “innovative payment and service delivery models to reduce program expenditures …while preserving or enhancing the quality of care” for those individuals who receive Medicare, Medicaid, or Children's Health Insurance Program (CHIP) benefits [31]. For Medicare-eligible ex-offenders, integrated service delivery models such as Accountable Care Organizations (ACOs), supported through the CMS Innovation Center, will offer coordinated high quality care [32]. Additionally, the CMS Innovation Center is testing Health Care Innovation projects in communities across the nation that aim to deliver better health, improved care and lower costs to people enrolled in Medicare, Medicaid and the Children's Health Insurance Program (CHIP), particularly aimed at those with the highest health care needs. One example includes the Transitions Clinic Network. For a project period of three years, the San Francisco Community College District (City College of San Francisco), in partnership with the University of California San Francisco and Yale University, will target the health care needs of high-risk/high-cost Medicaid and Medicaid-eligible patients released from prison, connecting them to care in 11 community health centers in six states, the District of Columbia, and Puerto Rico. The program will work with Departments of Correction to identify patients with chronic medical conditions prior to release and will use community health workers trained by the City College of San Francisco, who are ex-offenders themselves, to help these individuals navigate the care system, find primary care and other medical and social services, and coach them in chronic disease management [33].

Because of these efforts, ex-offenders may have increased opportunities to access coordinated care teams equipped to address and ameliorate their co-occurring morbidities. Integrated health care models, such as that proposed by the City College of San Francisco, have the potential to reduce disparities and improve outcomes such as reliance on emergency room care, fewer hospital admissions, and lower cost, with improved patient health and better access to appropriate care. Reentrants participating in integrated health care reported reductions in hospitalizations and time spent in hospitals [34]. Additionally, ex-offenders with co-occurring conditions who are linked to post-incarceration integrated health care remain in care and possess lower recidivism rates [35],[36].

Although the ACA provides opportunities to improve health and reduce health disparities through increased accessibility to health care services, assistance to maximize the benefits of these opportunities remains critical. Discharge planning is an important vehicle to ensure that soon to be released individuals are connected to care. Health discharge planning provides an opportunity to assess an offender's health needs, develop a disease treatment and management plan to be implemented upon release, and assist with documentation needed to access services. Health discharge planning has been typically conducted with inmates affected by HIV/AIDS or severe mental illness [37],[38]. However, pre-release health preparation is associated with improved rates of health insurance enrollment and subsequent utilization of health services [39],[40] and can been extended to individuals with a broader set of chronic health and behavioral conditions.

The incorporation of health insurance enrollment assistance and linkage to local integrated health systems into standard discharge planning policy for jails and prisons could be pivotal to continuity of health care and serve as a mechanism to reduce health disparities for those with a history of incarceration. Though Medicaid enrollment assistance is common practice in discharge planning for those with severe mental illness among U.S. state prison systems [41], assistance with Health Insurance Marketplace applications prior to release for all inmates can be adopted in this era of health reform. CMS has provided information on ways correctional systems can assist soon-to-be released inmates to engage in the Marketplace. The document suggests educating staff, inmates and inmates' families on opportunities to enroll in Medicaid or private health insurance, providing enrollment assistance, and sharing ideas and successful experiences with other correctional systems [42]. Additionally, relationships between correctional facilities and local integrated health systems would likely aid the transition to community care. Community providers can engage with future patients prior to release by assisting in health discharge planning and conducting health education and promotion activities to prepare the individual for self-maintenance and community care.

## 6. Conclusions

The Affordable Care Act offers opportunities to address health disparities, such as chronic health conditions and co-occurring mental health and substance use disorders, experienced by the reentry population that place them at risk for negative health outcomes and recidivism. Linking ex-offenders to care and services upon release increases the likelihood that they will remain in care and practice successful disease management. In addition to improving the overall health profile of this vulnerable population, ACA efforts will promote continuity of health care provided by correctional facilities and in the community. This may lead to decreased health disparities between those with a history of incarceration and those without and to a reduction in health disparities existing among ex-offenders based on race and ethnicity. Discharge planning provides an opportune moment to assist justice-involved individuals in attaining new health care opportunities provided by the ACA and set them on the trajectory for positive health outcomes as they reintegrate into society.
